# Underutilization of cervical cancer prevention services in low and middle income countries: a review of contributing factors

**DOI:** 10.11604/pamj.2015.21.231.6350

**Published:** 2015-07-30

**Authors:** Fresier Chidyaonga-Maseko, Maureen Leah Chirwa, Adamson Sinjani Muula

**Affiliations:** 1 School of Public Health and Family Medicine Health, University of Malawi, Mahatma Gandhi Road, Private Bag 360, Chichiri, Blantyre 3, Malawi; 2 Prime Health Consulting and Services, Prime Health Consulting and Services A47/5/240, Malingunde Road. P.O. Box 1926, Lilongwe, Malawi

**Keywords:** Cervical cancer, prevention, low and middle income countries

## Abstract

This review aims at identifying barriers to utilization of cervical cancer prevention services in low- and middle-income countries. An electronic search was conducted using the following key words, HPV vaccination, screening, barriers, utilization and low and middle income/developed countries. Using the Garrard (1999) Matrix method approach, a modified matrix was designed and used as a data collection tool and data related to each category listed on the tool were entered into a matrix containing columns reflecting the categories. Constant comparative analysis was used to identify thematic categories. 31 articles published between 2001 and 2014 were yielded from the search. Analysis of the contents of the articles showed that the underutilization of cervical cancer screening services in low and middle-income countries is the result of barriers in accessing and utilizing of the prevention services. Though not mutually exclusive, the barriers were categorized in three categories; individual, community and health system related. Individual barriers include lack of awareness and knowledge about risk factors and prevention of cervical cancer. Age, marital status, diffidence, social economic status, cultural and religious belief of the women also determine the women's' willingness to utilize the services. In some communities there is stigma attached to discussing reproductive health issues and this limits the young women's awareness of cervical cancer and its prevention. Understanding individual, community and health system barriers that hinder women's utilization of cervical cancer prevention services is very crucial in designing effective cervical cancer control programs in low- and middle-income countries.

## Introduction

Cervical cancer is one of the most common cancers among women of reproductive age in low- and middle-income countries(LMIC) [1]. According to The International Agency for Research on Cancer (IARC) report of December 2013, cervical cancer is the fourth most common cancer affecting women worldwide and most notable in low income countries of sub-Saharan Africa. Despite a drastic decrease in cervical cancer morbidity and mortality in high-income countries, there are 528000 new cases estimated globally every year [2]. It is also the fourth most common cause of cancer death in women worldwide. Every year more than 270000 women die from cervical cancer and more than 85% of these deaths are in low and middle income countries [3]. Almost every case of cervical cancer is potentially preventable [4] yet, in low- and middle-income countries, women have three times the risk of dying of cervical cancer compared to those in high-income countries [4]. A good approach to a comprehensive cervical cancer prevention and control is to act across the life course using the natural history of the disease to identify opportunities in relevant age groups to deliver effective interventions [3]. Thus cervical cancer prevention can be done at primary, secondary and tertiary levels. According to World Health Organization (WHO), primary prevention involves vaccinating girls between the ages of 9 to 13 with human papilloma virus (HPV) vaccine (Cevarix and Gardasil) and giving them appropriate health information and warning about the risk behaviors associated with cervical cancer [3–5]. Secondary prevention, ideally, involves early detection and treatment of subclinical, asymptomatic, or early disease in women of 30 years or older, without obvious signs or symptoms of cancer. Secondary cancer prevention includes identifying women who are at risk for developing malignancy and implementing appropriate screening recommendations based on the risk assessment [3, 5]. This may involve the use of cytology smears or, non-cytology based screening methods followed by treatment of the precancerous lesions [6]. The non-cytology based screening include the human papilloma virus DNA test and the visual inspection with acetic acid (VIA), which may be followed by cryotherapy to those women with positive test results. Both of these approaches perform as well as or better than cytology based screening for identifying high-grade cervical cancer precursor lesions [7]. Cryotherapy, on the other hand, is a relatively low technology treatment method that is highly efficacious and has minimal morbidity [7, 8]. Women with invasive cervical cancer need tertiary prevention which may involve ablative surgery, radiotherapy or chemotherapy regardless of age [3]. Even though the impact of screening has never been demonstrated through randomized trials, empirical evidence suggests that cervical cancer screening represents a viable strategy for significant reduction in morbidity and mortality in LMIC countries [9].

Attainment of high HPV vaccination, screening coverage rates and treatment of all women with precursor lesions in target groups is essential for any cervical cancer prevention program success, and is an immense challenge [10]. Most health systems in low- and middle-income countries have found it very challenging to come up with comprehensive cervical cancer prevention programs that can attain high coverage of cervical cancer HPV vaccination, screening and treatment. In 2005, a situation analysis of cervical cancer services in five health systems from countries in eastern, central, and southern Africa, conducted by Bradley and his colleagues, documented significant gaps in capacity. Many low and middle-income countries have had established cervical cancer prevention programs in operation for decades. Despite the existence of the programs, cervical cancer screening coverage is very low [11]. For instance in Malawi, despite having the cervical cancer prevention program offering free services in all public health facilities for more than two decades, the screening coverage is less than 5%, and is concentrated in urban and semi-urban areas. The questions are why should women keep on dying of cervical cancer when the disease is easily preventable? Why is there underutilization of cervical cancer prevention services in most low- and middle-income countries? This review aims at reviewing studies on factors that contribute to the low uptake of cervical cancer prevention services in low- and middle-income countries.

## Methods

To come up with this review, a six step process was undertaken. This involved formulation of the review questions, defining inclusion and exclusion criteria, developing search strategy, selecting studies, extracting ideas, analyzing and interpreting the results [12, 13].

### Search strategy

An electronic search was conducted using Boolean Operator AND, and combining the following key words: cervical cancer, prevention, HPV vaccination, screening, treatment, barriers, utilization, developing countries and low and middle income countries [14]. Since Prevention of cervical cancer covers HPV vaccine, screening and treatment, the key worlds were interchangeably used in the combinations and so were developing countries and low and middle income countries. The search was undertaken in both peer-reviewed and grey literature databases, which comprised: CancerLit, Cochrane Database of Systematic Reviews, EMBASE, Database of Abstract of Reviews of Effects (DARE), MEDLINE, Education Resource Information Centre (ERIC) Google scholar, Africa Journal Online and Proquest Dissertations and Theses. We also reviewed reference lists of selected articles to identify additional secondary articles not found during the online search.

### Inclusion and exclusion criteria

Based on the key questions, we came up with inclusion and exclusion criteria. Included were full articles written in English language and reporting on factors of underutilization of cervical cancer prevention services in low and middle income countries. Articles written before 2001 were excluded.

### Selection process

Based on the abstract, each article was screened to assess if it provided relevant information on barriers to utilization of cervical cancer prevention services. Those that had full articles available were then entered into an Endnote library.

### Data collection

Using the Garrard (1999) Matrix method approach, a modified matrix was designed and used as a data collection tool [15–17] as shown in Table 1. The tool consisted of categorical columns in which data related to the author, year of publication, category and evidence on barriers to cervical cancer prevention service utilization were later entered. After careful reading of the article, data related to each category listed on the tool were entered into a matrix containing columns reflecting the categories. Constant comparative analysis was conducted to identify thematic categories.

**Table 1:** Modified garrard matrix

Author(s)	Year	Barrier	Category
Hoque ME, Ghuman S, Coopoosmay R, Van Hal G	2014	cervical cancer knowledge had a significantly negative relationship with barriers to cervical cancer screening	Individual related barrier
Jia Y, Li S, Yang R, Zhou H, Xiang Q, Hu T, Zhang Q, Chen Z, Ma D, Feng L	2013	Knowledge of cervical cancer and utilization of the services. Socio-economic status and cervical cancer prevention	
Banura C, Mirembe FM, Katahoire AR, Namujju PB, Mbidde EK	2012	Awareness and HPV vaccination. Concerns about future fertility of the vaccinated girls	
Mingo AM, Panozzo CA, Diangi YT, Smith JS, Steenhoff AP, Ramogola-Masire D, Brewer NT	2012	Age, income and awareness and cervical cancer prevention services utilization among Batswana women.	
Ma J, et.al	2012	Level of woman's education and cervical cancer prevention	
Urasa M, Darj E	2011	Awareness of cervical cancer and its cause	
LaMontagne DS, *et al*	2011	Awareness and HPV vaccination. Concerns about future fertility of the vaccinated girls	
Carr KC, Sellors JW	2004	Lack of awareness about cervical cancer	
Alliance for Cervical Cancer Prevention	2004	one's geographic and economic barriers to the services	
Wong LP, Wong YL, Low WY, Khoo EM, Shuib R	2008	Association of HPV and cervical cancer. Marital status and screening.	
Hilton S, Hunt K	2010	Association of HPV and cervical cancer	
Moreira ED, Jr., Oliveira BG, Ferraz FM, Costa S, Costa Filho JO, Karic G	2006	Awareness of cervical cancer and its association with human papilloma virus infection	
Markovic M, Kesic V, Topic L, Matejic B	2005	Women's poor knowledge of the existence and availability of screening, socio-cultural beliefs about preventive health care, gender roles, inadequate public health education	
Matejic B et.al	2011	Socio-economic status and cervical cancer prevention	
Pelcastre-Villafuerte B, et.al	2007	Fear of abandonment by partners	
Sauvageau C, et.al	2007	Socio-economic status and cervical cancer prevention	
Tsua VD, Pollack AE	2005	Fear associated with knowing that one has cervical cancer	
McFarland DM	2003	Inadequate knowledge about the testing and limited access to the services.	
White HL, Mulambia C, Sinkala M, Mwanahamuntu MH, Parham GP, Moneyham L, Grimley DM, Chamot E	2012	Stigma associated with being diagnosed with cervical cancer	Community related factors
Wong VS, Kawamoto CT	2010	husbands preventing women to go for screening	
Al-Naggar R.A, Low W.Y , Md IZ	2010	Screening tests break one's virginity	
Thomas. V. N, Saleem. T, Abraham.R	2005	comfortable to be seen naked by a male physician	
Arlene C	2005	Machismo and Gender roles	
Bingham A, Bishop A, Coffey P, Winkler J, Bradley J, Dzuba I, Agurto I	2003	Perception that cervical cancer is related with HIV as such fear of stigma associated to it	
Hutubessy R, Levin A, Wang S, Morgan W, Ally M, John T, Broutet N	2012	Exorbitant prices of HPV vaccines	Health system related factors
Sankaranarayanan.R	2009	Dosing of HPV vaccines	
Tsu VD, Levin CE	2008	User fees and the lack of reasonable health care insurance	
Agurto I, Bishop A, Sanchez G, Betancourt Z, Robles S	2004	inability of the health system to provide high-quality services	
Alliance for Cervical Cancer Prevention	2004	Transportation to cervical cancer clinics	
Sankaranarayanan .R, Budukh. A. M, Rajkumar. R	2001	Adaquatefinancial resources, infrastructure, and trained manpower, and elaborates surveillance mechanisms for screening, investigating, treating, and follow-up of the targeted women	
Chirenje, M. Z., S. Rusakaniko, L. Kirumbi, W. E.et. al	2001	Long distances and cost of sending the smear to the processing centres	

## Current status of knowledge

**Figure 1:**
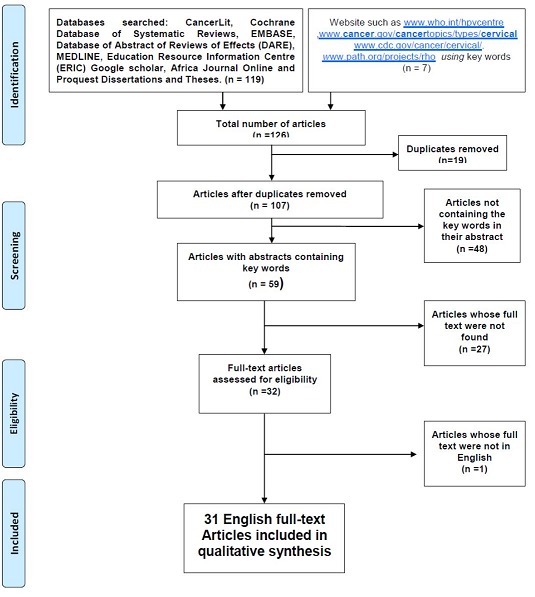
Search strategy flow diagram

The final screening resulted in a sample of 31 full text articles that had information on barriers to women' utilization of cervical cancer prevention services in low- and middle- income countries. These were articles published between 2001 and 2014. Figure 1 illustrates the search strategy and the selection process [18]. The majority of the papers were published after 2008 and there were no papers published in 2002. After constant comparative analysis of the data, three themes on barriers to utilization of cervical cancer prevention services were identified. Although not mutually exclusive, the results shows that women in low- and middle-income countries fail to utilize cervical cancer prevention services due to factors related to individual, community and health systems Figure 1.

### Individual-related barriers

Women and young girls in low and middle income countries face many barriers that prevent them from receiving adequate and timely cervical cancer vaccination, screening and treatment. Lack of awareness about cervical cancer and knowledge about prevention are key factors [19]. In most low and middle income countries, barriers to cervical cancer prevention services uptake include lack of or inadequate knowledge about the disease, lack of familiarity with the concept of preventive health care and one's geographic and economic inaccessibility to the services [20] There is limited knowledge about cervical cancer, especially the association with human papillomavirus [21]. Surveys have shown that awareness about the human papilloma virus, which is known to be the major cause of cervical cancer, is very low [21, 22]. In a study carried out to assess nurses' awareness of cervical cancer and their own screening practices at a regional hospital in Tanzania, over 60% of the nurses did not know that cervical cancer was a human papilloma virus infection [23]. This was also the case in a qualitative study which used face-to-face in-depth interviews to investigate knowledge, attitudes and beliefs on cervical cancer screening among Malaysian women. It was noted that women had poor knowledge about cervical cancer and how it is prevented [21]. These findings were also in line with the findings of a study that was conducted in Brazil by Moreira and colleagues. Sixty seven percent of the women in this study did not know that HPV causes cervical cancer [24]. Markovic and colleagues argued that in the absence of adequate knowledge, women are not likely to present for screening, or might do so at a stage when cervical cancer can no longer be prevented or effectively treated. Participants in this study reported that their individual ignorance was influenced by socio-cultural factors and that it did affect their utilization of cervical cancer prevention services [25]. The higher the woman's knowledge about cervical cancer, the more willing were women to utilize cervical cancer prevention services. A survey carried in a low income county of Wufeng in central China that aimed at determining women's knowledge about cervical cancer and screening, demographic characteristics and the barriers to screening showed that women who had knowledge of cervical cancer were more willing to utilize the services than those without knowledge [26]. In an HPV vaccine demonstration project in India, Peru, Uganda and Vietnam, parents and guardians of girls who were partially vaccinated or who did not get vaccinated mentioned lack of awareness about the vaccination program as the reason for not being vaccinated. In South Africa, a cross sectional study which was conducted among university women to elicit information about knowledge and beliefs, and screening history showed that cervical cancer knowledge had a significantly negative relationship with barriers to cervical cancer screening [27]. In Uganda cultural barriers are a concern to HPV vaccination. It was noted that parents in communities that participated in the HPV demonstration project were concerned about future fertility of the vaccinated girls [4, 28].

Marital status can also determine a woman's utilization of cervical cancer prevention services, especially when it comes to screening. In the Malaysian study, it was found out that 50% of the women did not recognize cervical cancer risk factors. Married women had a higher recognition of cervical cancer risk factors than did those who have never been married [21]. This is consistent with the finding from two studies that were conducted in Botswana by Mingo and McFarland [29, 30]. In Mingo's study, it was found that the women who were older and had higher income or had heard of cervical cancer were more likely to utilize cervical cancer prevention services than young women and those with little income or those who had never heard about cervical cancer [29]. Similarly in McFarland's study, it was noted that participants in the low income category had little knowledge of cervical cancer and Pap smear testing and their utilization of cervical cancer prevention services were also low. The study showed that major barriers to cervical cancer screening with Pap smear included inadequate knowledge about testing and limited accessibility to the services [30]. Lack of awareness about cervical cancer and how to prevent it, is an important obstacle to improving screening coverage. Fear of learning that one has cervical cancer and fear that cervical cancer is associated with sexually transmitted infections (STI) is a potential barrier for a woman to go for screening and treatment [31] as there is stigma associated with STIs [32]. A study conducted among Zambian women attending a cervical cancer screening program showed that women perceived cervical cancer to be associated with social stigma. This is because of its location, dire natural course and its connections to socially-condemned behaviors which are associated with HIV and AIDS. Women with such perceptions may not go for cervical cancer screening [32, 33] Social economic status (SES) in particular education plays a very crucial role in the women's knowledge about risks, prevention and management of cervical cancer [34]. It is documented that social economic status of a woman in society is the most prominent factor influencing the woman's presentation to cervical cancer screening [35]. Lower cervical cancer screening rates and negative attitudes toward cervical cancer screening are more common in lower socioeconomic groups. In Belgrade (Serbia), a study conducted in 2010, showed that women with lower economic status were less likely to undergo cervical cancer screening even for freely available screening services [35]. It was reported that social economic factors inhibit women from utilizing cervical cancer prevention services, thereby resulting in increased cervical cancer morbidity and mortality [36]. In the Wofeng study, women with higher education and income had higher levels of knowledge about cervical cancer and were much more willing to go for cervical cancer screening than those who had less education and income [26].

In some cases women were reported to fail going for cervical cancer prevention screening because of diffidence (state of shyness) and fear [37]. This was confirmed in an operational research that was conducted in Madagascar, Malawi, Nigeria, Uganda, Tanzania and Zambia by WHO, which aimed at assessing the acceptability and feasibility of implementing a cervical cancer prevention program with “see to treat” approach based on VIA and cryotherapy. In this study, shyness and fear as reasons for refusing to be screened for cervical cancer were mentioned by 3% and 51% of the 75 women interviewed, respectively. In a study conducted among women in Chuuk state, federal state of Micronesia, in which Wong and Kawamoto wanted to understand cervical cancer prevention and screening in Chuukese women, women reported that they did not want to participate in cervical cancer screening because culturally, they did not want to show their genitals to anyone else apart from their husbands. They stated that showing private areas to others is disrespectful and they felt embarrassed if someone else, including doctors, saw their genital. On the other hand, some husbands do not want doctors to see private parts of their wives. The women also did not want male doctors to examine them and stated that often times, the husbands would not allow them to go for screening [38], which limits their cervical cancer screening uptake. It was also noted that women were so afraid of the cervical cancer diagnosis that they would rather die than know that they had cervical cancer. In some cultures, cancer in general is considered to be a death sentence. A study which focused on social construction of cervical cancer screening among women in Panama City reported that Hispanic women conceptualized cancer as representing a death sentence. The study also reported that women with strong religious background do have the belief of divine predestination called fatalism. Accordingly, with this belief, God's fate or will destines certain individuals to develop illnesses such as cervical cancer [39]. This type of belief prevents women across social classes from seeking screening services because they think the disease is beyond their control. Similar sociocultural barriers are also evident in HPV vaccination. Parental fear of future side effect such as infertility, increased and/or earlier sexual activity and the safety of the vaccine in LMIC have made girls to shun away from HPV vaccination.

### Community-related factors

Sexuality is identified as a taboo topic for parents who want to protect family reputation and encourage modesty, particularly among daughters. This results in young women not having the necessary sexual health education [25]. In a study conducted by Markovic (2005) in central Serbia that explored women's cervical cancer-screening behaviors, participants viewed a lack of informal sexual education to be a barrier to cervical cancer screening. Participants also mentioned that there is stigma attached to discussing reproductive health issues in their communities [25], which contributes to the women having little knowledge about cervical cancer and its prevention. Among black and minority ethnic communities in the research study conducted by Thomas et.al, it was also reported that African communities never talked about some cancers, especially cancer of the cervix as it is regarded to be taboo [40]. Gender roles and their overall subordinate position in the family and society influence women's poor ability to access cervical cancer screening [25]. In a study conducted in Mexico, which aimed at attempting to analyze the role of several social and cultural factors in relation to the early detection of cervical cancer, it was reported that women feared abandonment by their partners when faced with confirmation of diagnosis of cervical cancer. The study mainly focused on the influence of partner and the social networks regarding utilization of the Pap test [41]. Such fear would make women not to go for cervical cancer screening.

### Health system-related factors

A well-organised cervical cancer prevention service takes into consideration adaquate financial resources, infrastructure, and trained human resources, and elaborates surveillance mechanisms for screening, investigating, treating, and follow-up of the targeted women. Focus is also on what screening test to use [6]. In the 2005 Markovic study, participants criticized the way that health care providers offer cervical cancer prevention services can also determine utilization of the cervical cancer prevention services. In the focus group discussions, women mentioned that access to the health personel was problematic for them in both towns and the outer metropolitan surbub [25]. Bingham et al, in an article that summarized the experiences of research studies in Bolivia, Peru, kenya, South Africa and Mexico, reported that for some women, especially those living in communities where there was minimal access to health care, the location of the service facility is an important determinant of participation in cervical cancer screeningand treatment programs. Geographic inaccessibility remains a central barrier in most resource-poor settings, because a significant portion of the population at risk for cervical cancer might be located in areas where little or no coverage currently exists [33]. In Peru, for instance, the researcher representing the Alliance for Cervical Cancer Prevention (ACCP) found that screening rates were much lower in districts where services were distant or difficult to access. The ACCP program researcher also noted that regional coverage rates were much higher where static services were more accessible to major population centres or where mobile campaigns brought services to women [19, 33]. On the other hand, studies conducted by the ACCP in Mexico and western Kenya, women reported that transportation cost and distance played a significant role in screening participation. In some rural areas that were under the ACCP program, women had to hire a taxi to go for screening because there were no public transport [33, 42]. This impeded the women from participating in the cervical cancer prevention programs. Kenyan studies also showed that women were unable to attend the cervical cancer prevention service because they had to travel up to eight hours and at an average cost of a day's agricultural wage to access the services [33]. A study that aimed at determining factors that influence cervical cancer diagnosis and treatment in the countries of East, Central and Southern Africa (ECSA), reported that absence or frequent shortage of medical supplies and drugs needed to screen and to treat the disease is also an important barrier to cervical cancer prevention services utilization in the ECSA region. In those health systems that use cytological method of screening, the long distances and cost of sending the smear to the process centres also becomes a challenge to provision of cervical cancer screening in some geographical areas of the system [43].

Availability of appropriate personnel at the health facility to provide cervical cancer prevention services also contribute to women's utilization of the services. In the Markovic study, it was reported that women complained that sometimes when they go to a health facility for cervical cancer screening, there are no health personnel to offer them the particular service they want. Sometimes, even when the appropriate health personel is available, the time women wait to be assisted is long. The women reported that they can wait up to four hours before being seen by medical personnel and, in some cases, the service providers are absent from work for the whole day [25]. Qualitative studies on barriers and benefits of cervical cancer prevention services in Latin American countries conducted by ACCP reported that most women experienced anxiety while waiting for the test results, which contributed to their overall fear of cancer and, as such, refrain from using the services [20]. The way the health care providers provide the cervical cancer prevention services can impede women from demanding services. The client-provider relationship affects a client's level of satisfaction on a service. Bingham and her colleagues gave examples of the conditions under which counseling takes place, which can make a woman not to come for the services. The paper points out that the way the provider communicates information to the women, the flexibility of the provider to take question from the women, the process of informed consent, and the respect for privacy and confidentiality are some of the contributing factors that would influence women's experience [33] with cervical cancer prevention services that might make them not to return for the service. Brusque behavior by cervical cancer service providers have also been reported to influence women not to utilize cervical cancer prevention services. From the study that was conducted in Peru, Mexico, Kenya and South Africa, it was noted that women would not patronize cervical cancer prevention services that were delivered by a provider who does not take time to converse with them, answer their questions, explain procedures, and give them encouragement [33]. In the Markovic study, one woman reported not to utilize the services because doctors did not respect the women sufficiently and also that the waiting rooms were crowded [25]. Health system related factors have also contributed to underutilization of HPV vaccine. In the HPV vaccine demonstration study which was carried in India, Peru, Uganda and Viet Nam, the main barriers to vaccination were girls being absent from school on the day of vaccination, the failure of the health system to provide sufficient information about cervical cancer and difficulty in determining a girl's eligibility [28].

In those health systems, where health care is not free at a point of delivery, accessing cervical cancer prevention services is not easy for some women due to the prohibitive costs of the services for both the woman and her family [42]. User fees and the lack of reasonable health care insurance have led women not to utilize cervical cancer prevention services in low- and middle-income countries, as reported in the article, “preventing cervical cancer in low-resource settings: How far have we come and what does the future hold?” [44]. This is also the case with HPV vaccines uptake. In most low and middle income countries, the HPV vaccine price offered to the public sector ranges from US$15 to more than US$130 per dose [45] which most families cannot afford to pay for their girls. On the other hand the logistics of the HPV vaccine itself poses a challenge to girls. The vaccine is supposed to be administered in three doses over a period of six month [46]. This can discourage parents or guardians to take girls for vaccination or completing taking all the three needed doses. Studies from low- and middle-income countries have shown that, common health system barriers to cervical cancer service delivery included the inability of the system to make the services accessible to the women and inability of the health system to provide high-quality services [47]. On the other hand provision of services that lack comfort and privacy in the facilities, discourtesy on the part of facility staff and prohibitive cost of services have also known to contribute negatively to utilization of cervical cancer prevention services [20, 47].

## Discussion

Literature suggests that some women and girls in low and middle income countries do not utilize cervical cancer prevention services due to individual, community and health systems related factors. One of the key factors is lack of awareness about cervical cancer and how it can be prevented. Poor knowledge about the disease might be caused by the women's low levels of education but may also arise from the failure of the health system to provide women with adequate information about cervical cancer. Women who are more educated and also those with more income are very likely to utilize cervical cancer prevention services than those with little or no education and income. This is not surprising as we expect those women who are educated to have an understanding of the cause, risk factors, prevention mechanism and treatment of the disease and as such are able to demand the services. On the other hand, those with more income are in a better position to purchase the costly preventive services than those with less or no income. With low level of education and little knowledge about cervical cancer, women in low and middle income countries attach stigma to cervical cancer or relate cervical cancer to HIV/AIDS. With the right information through health education, women have a good understanding of cervical cancer which can help them overcome their fears and cultural beliefs about the disease. Inability of health systems to provide high-quality services have also been identified as a barrier to utilizing cervical cancer prevention services. This is mostly due to lack of resources such as infrastructure, manpower and medical supplies and equipment necessary to implement effective cervical cancer interventions.

### Limitations of the review

The study has a number of limitations. We only included articles and reviews written in English language and published from 2001 to 2013 and the search was limited to online sources. Inclusion of studies from other languages published even before 2001 would have added more information to the subject matter.

## Conclusion

Screening is the key component of secondary prevention of cervical cancer because it is the only way to detect neoplasia grades 2 or 3, which are considered to be the true precancerous lesion. Understanding individual, community and health system barriers that hinder women's utilization of cervical cancer prevention services is essential to national health systems in low and middle income countries. The information is important in designing effective cervical cancer control programs that can attract more women for screening and treatment. It is therefore imperative that health systems in low and middle income countries should come up with health policies that are needed to facilitate development and implementation of effective cervical cancer prevention programs. Programs that locally and appropriately designed to reduce barriers to screening such as poor quality health resources, economic and social inaccessibility, lack of awareness about preventing cervical cancer, difficulties in paying for services and social stigma associated with cervical cancer [48].

## References

[1] Setoodeh R, Hakam A, Shan Y (2012). Cerebral metastasis of cervical cancer, report of two cases and review of the literature. Int J Clin Exp Pathol..

[2] The International Agency for Research on Cancer (2013). Latest world cancer statistics Global cancer burden rises to 14.1 million new cases in 2012: Marked increase in breast cancers must be addressed.

[3] World Health Organization Comprehensive cervical cancer prevention and control:a healthier future for girls and women. WHO Guidance Note.

[4] Banura C, Mirembe FM, Katahoire AR, Namujju PB, Mbidde EK (2012). Universal routine HPV vaccination for young girls in Uganda: a review of opportunities and potential obstacles. Infect Agent Cancer..

[5] Mariani L, Pagliusi S (2008). Vaccination and screening programs: harmonizing prevention strategies for HPV-related diseases. J Exp Clin Cancer Res..

[6] Sankaranarayanan R, Budukh AM, Rajkumar R (2001). Effective screening programmes for cervical cancer in low- and middle-income developing countries. Bull World Health Organ..

[7] Denny L, Kuhn L, De Souza M, Pollack AE, Dupree W, Wright TC (2005). Screen-and-treat approaches for cervical cancer prevention in low-resource settings: a randomized controlled trial. JAMA..

[8] Bradley J, Barone M, Mahe C, Lewis R, Luciani S (2005). Delivering cervical cancer prevention services in low-resource settings. Int J Gynaecol Obstet..

[9] Gakidou E, Nordhagen S, Obermeyer Z (2008). Coverage of Cervical Cancer Screening in 57 Countries: Low Average Levels and Large Inequalities. PLoS Med..

[10] Kawonga M, Fonn S (2008). Achieving effective cervical screening coverage in South Africa through human resources and health systems development. Reproductive Health Matters..

[11] Kathy Shapiro, Emma Ottolenghi, Patricia Claeys, Petitpierre J, World Health Organisation (2006). Comprehensive Cervical Cancer Control A guide to essential practice.

[12] Uman LS (2011). Systematic Reviews and Meta-Analyses. Journal of the Canadian Academy of Child and Adolescent Psychiatry..

[13] Ganong LH (1987). Integrative reviews of nursing research. Research in Nursing & Health..

[14] Lu M, Moritz S, Lorenzetti D, Sykes L, Straus S, Quan H (2012). A systematic review of interventions to increase breast and cervical cancer screening uptake among Asian women. BMC Public Health..

[15] Garrard J (2011). Health Sciences Literature Review Made Easy: the Matrix Method. Sudbury: Jones & Bartlett Learning..

[16] Klopper R, Lubbe S, Rugbeer H (2007). The Matrix Method of Literature Review. Alternation..

[17] Pongjaturawit Y, Harrigan RC (2003). Parent participation in the care of hospitalized child in Thai and Western cultures. Issues in comprehensive pediatric nursing..

[18] Moher D, Liberati A, Tetzlaff J, Altman DG (2009). Preferred reporting items for systematic reviews and meta-analyses: the PRISMA statement. PLoS Med..

[19] Carr KC, Sellors JW (2004). Cervical cancer screening in low resource settings using visual inspection with acetic acid. J Midwifery Womens Health..

[20] Allliance for Cervical Cancer Prevention (2004). Improving Screening Coverage Rates of Cervical Cancer Prevention Programs: A Focus on Communities. In: Cervical Cancer Prevention Issues in Depth.

[21] Wong LP, Wong YL, Low WY, Khoo EM, Shuib R (2008). Cervical cancer screening attitudes and beliefs of Malaysian women who have never had a pap smear: a qualitative study. Int J Behav Med..

[22] Hilton S, Hunt K (2010). Coverage of Jade Goody's cervical cancer in UK newspapers: a missed opportunity for health promotion?. BMC Public Health..

[23] Urasa M, Darj E (2011). Knowledge of cervical cancer and screening practices of nurses at a regional hospital in Tanzania. Afr Health Sci..

[24] Moreira ED, Oliveira BG, Ferraz FM, Costa S, Costa Filho JO, Karic G (2006). Knowledge and attitudes about human papillomavirus, Pap smears, and cervical cancer among young women in Brazil: implications for health education and prevention. Int J Gynecol Cancer..

[25] Markovic M, Kesic V, Topic L, Matejic B (2005). Barriers to cervical cancer screening: A qualitative study with women in Serbia. Social Science & Medicine..

[26] Jia Y, Li S, Yang R, Zhou H, Xiang Q, Hu T, Zhang Q, Chen Z, Ma D, Feng L (2013). Knowledge about cervical cancer and barriers of screening program among women in Wufeng County, a high-incidence region of cervical cancer in China. PLoS One..

[27] Hoque ME, Ghuman S, Coopoosmay R, Van Hal G (2014). Cervical Cancer Screening among University Students in South Africa: A Theory based Study. PLoS one..

[28] LaMontagne DS, Barge S, Le NT, Mugisha E, Penny ME, Gandhi S, Janmohamed A, Kumakech E, Mosqueira NR, Nguyen NQ (2011). Human papillomavirus vaccine delivery strategies that achieved high coverage in low- and middle-income countries. Bulletin of the World Health Organization..

[29] Mingo AM, Panozzo CA, Diangi YT, Smith JS, Steenhoff AP, Ramogola-Masire D, Brewer NT (2012). Cervical Cancer Awareness and Screening in Botswana. Int J Gynecol Cancer..

[30] McFarland DM (2003). Cervical cancer and Pap smear screening in Botswana: knowledge and perceptions. Int Nurs Rev..

[31] Tsua VD, Pollack AE (2005). Preventing Cervical Cancer in Low-Resource Settings: How Far have we Come and what does the Future Hold?. International Journal of Gynecology and Obstetrics..

[32] White HL, Mulambia C, Sinkala M, Mwanahamuntu MH, Parham GP, Moneyham L, Grimley DM, Chamot E (2012). “Worse than HIV” or “not as serious as other diseases?” Conceptualization of cervical cancer among newly screened women in Zambia. Social Science & Medicine..

[33] Bingham A, Bishop A, Coffey P, Winkler J, Bradley J, Dzuba I, Agurto I (2003). Factors affecting utilization of cervical cancer prevention services in low-resource settings. Salud Publica Mex..

[34] Ma J, Zhu Q, Han S, Zhang Y, Ou W, Wang H, Zhao J, Liu Z (2012). Effect of socio-economic factors on delayed access to health care among Chinese cervical cancer patients with late rectal complications after radiotherapy. Gynecologic Oncology..

[35] Matejic B, Vukovic D, Pekmezovic T, Kesic V, Markovic M (2011). Determinants of preventive health behavior in relation to cervical cancer screening among the female population of Belgrade. Health Education Research..

[36] Sauvageau C, Duval B, Gilca V, Lavoie F, Ouakki M (2007). Human Papilloma Virus vaccine and cervical cancer screening acceptability among adults in Quebec, Canada. BMC Public Health..

[37] http://www.unfpa.org/webdav/site/global/shared/events/Cervical%20Cancer%20Event%202010/Nathalie%20Broutet%20-%20WHO%20six%20Afr%20country%20study%20%5BCompatibility%20Mode%5D.pdf.

[38] Wong VS, Kawamoto CT (2010). Understanding cervical cancer prevention and screening in Chuukese women in Hawaii. Hawaii Med J..

[39] Arlene C (2005). Social construction of cervical cancer screening among women in Panama City, Panama. University of South Florida.

[40] Thomas VN, Saleem T, Abraham R (2005). Barriers to effective uptake of cancer screening among black and minority ethnic groups. International Journal of Palliative Nursing..

[41] Pelcastre-Villafuerte B, Tirado-Gomez L, Mohar-Betancourt A, Lopez-Cervantes M (2007). Cervical cancer: a qualitative study on subjectivity, family, gender and health services. Reproductive Health..

[42] Alliance for Cervical Cancer Prevention (2004). Planning and Implementing Cervical Cancer Prevention and Control Programs: A Manual for Managers. Cervical Cancer Prevention Fact Sheet.

[43] Chirenje MZ, Rusakaniko S, Kirumbi L, Ngwalle WE, Makuta-Tlebere P, Kaggwa S, Mpanju-Shumbusho W, Makoae L (2001). Situation analysis for cervical cancer diagnosis and treatment in East, Central and Southern African countries. Bull World Health Organ..

[44] Tsu VD, Levin CE (2008). Making the case for cervical cancer prevention: what about equity?. Reproductive Health Matters..

[45] Hutubessy R, Levin A, Wang S, Morgan W, Ally M, John T, Broutet N (2012). A case study using the United Republic of Tanzania: costing nationwide HPV vaccine delivery using the WHO Cervical Cancer Prevention and Control Costing Tool. BMC medicine..

[46] Sankaranarayanan R (2009). HPV vaccination: the promise & problems. The Indian journal of medical research..

[47] Agurto I, Bishop A, Sanchez G, Betancourt Z, Robles S (2004). Perceived barriers and benefits to cervical cancer screening in Latin America. Prev Med..

[48] Nene B, Jayant K, Arrossi S, Shastri S, Budukh A, Hingmire S, Muwonge R, Malvi S, Dinshaw K, Sankaranarayanan R (2007). Determinants of women's participation in cervical cancer screening trial, Maharashtra, India. Bull World Health Organ..

